# Enhancing the Hydrophilicity and Cell Attachment of 3D Printed PCL/Graphene Scaffolds for Bone Tissue Engineering

**DOI:** 10.3390/ma9120992

**Published:** 2016-12-07

**Authors:** Weiguang Wang, Guilherme Caetano, William Stephen Ambler, Jonny James Blaker, Marco Andrey Frade, Parthasarathi Mandal, Carl Diver, Paulo Bártolo

**Affiliations:** 1 Manchester Institute of Biotechnology, School of Mechanical, Aerospace and Civil Engineering, University of Manchester, Manchester M13 9PL, UK; weiguang.wang@postgrad.manchester.ac.uk (W.W.); caetanogf@gmail.com (G.C.); partha.mandal@manchester.ac.uk (P.M.); carl.diver@manchester.ac.uk (C.D.); 2 Department of Internal Medicine, Ribeirão Preto Medical School, University of São Paulo (USP), Ribeirão Preto, SP 14049-900, Brazil; prof.marcoandrey@gmail.com; 3 Bio/Active Materials Group, School of Materials, The University of Manchester, Manchester M13 9PL, UK; william.ambler@postgrad.manchester.ac.uk (W.S.A.); jonny.blaker@manchester.ac.uk (J.J.B.)

**Keywords:** biofabrication, composite materials, graphene, hydrophilicity, polycaprolactone, scaffolds, surface modification, tissue engineering

## Abstract

Scaffolds are physical substrates for cell attachment, proliferation, and differentiation, ultimately leading to the regeneration of tissues. They must be designed according to specific biomechanical requirements, i.e., certain standards in terms of mechanical properties, surface characteristics, porosity, degradability, and biocompatibility. The optimal design of a scaffold for a specific tissue strongly depends on both materials and manufacturing processes, as well as surface treatment. Polymeric scaffolds reinforced with electro-active particles could play a key role in tissue engineering by modulating cell proliferation and differentiation. This paper investigates the use of an extrusion-based additive manufacturing system to produce poly(*ε*-caprolactone) (PCL)/pristine graphene scaffolds for bone tissue applications and the influence of chemical surface modification on their biological behaviour. Scaffolds with the same architecture but different concentrations of pristine graphene were evaluated from surface property and biological points of view. Results show that the addition of pristine graphene had a positive impact on cell viability and proliferation, and that surface modification leads to improved cell response.

## 1. Introduction

Three-dimensional (3D) scaffolds fabricated by additive manufacturing are a promising strategy in tissue engineering for the replacement and regeneration of damaged tissue. Such scaffolds should ideally be stimulatory, as well as biocompatible, degradable, and designed according to specific requirements to create a highly porous structure with interconnected pores [1,2,3]. These characteristics can provide an appropriate environment for cells and play an important role as a physical substrate for cell attachment, proliferation, and differentiation, as well as integration to the host tissue in order to regenerate the defect [4,5,6]. Although some new methods using shape memory materials, such as bioprinting and 4D printing, are under development [7,8,9,10], they are very much at infancy and less mature than scaffold technology.

An ideal approach is to combine porous scaffolds with living progenitor cells, especially for elderly people whose cell growth and cell differentiation are age-compromised. Cellularised scaffolds, as previously reported using human adipose-derived mesenchymal stem cells (ADSCs) in a bone regeneration animal model [11], might stimulate the tissue around the damage area towards regeneration whilst playing an important role to support cell migration, cell attachment, proliferation, and differentiation.

Achieving suitable cell attachment to the scaffold is key to success; however, it is challenging with highly hydrophobic scaffold matrices, which may result in inefficient cell colonisation. Material surface modification such as plasma, laser and chemical treatment, and protein coating is commonly used in order to improve cell attachment, leading to more efficient scaffold colonisation [12,13,14,15,16,17]. However, some of these techniques are expensive, require long times, and in some cases are non-reproducible [12,13,14].

Pristine graphene, a two-dimensional carbon nanofiller, could play an important role in enhancing polymer material properties because it can improve solubility, processability, and mechanical and conductivity properties. Furthermore, it is suggested that graphene composites are able to provide a dramatic improvement in these properties with very low filler content [18,19,20,21,22]. Controversial discussion about graphene usage to develop and produce biomaterials has been reported in terms of cytotoxicity. Some research works have presented graphene-based composites as materials that might have potential cytotoxicity risks [23,24,25], while other studies report good cytocompatibility and the ability to stimulate cell proliferation, for example, with graphene coatings [26,27].

In order to enhance cell attachment and biological performance of poly(*ε*-caprolactone) (PCL) scaffolds, this paper investigates the addition of three different small concentrations of pristine graphene, as well as a simple sodium hydroxide (NaOH) surface treatment, to render the scaffolds more hydrophilic. Two major effects were considered: the effect of pristine graphene in small concentrations on the cell viability/proliferation rate and the effect of NaOH chemical treatment on the surface properties such that the hydrophobic feature of PCL scaffolds is changed.

## 2. Results and Discussion

### 2.1. Surface Modification Evaluated by Contact Angle

**Figure 1:**
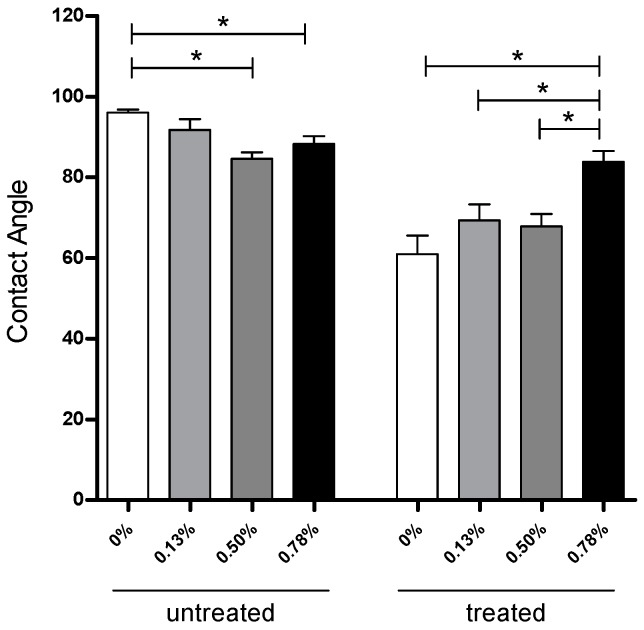
Summary of the apparent water-in-air contact angle for scaffolds containing different pristine graphene concentrations untreated and treated with NaOH 5M. * Statistical evidence (*p* < 0.05) analysed with a one-way ANOVA and Tukey’s post-hoc test.

The apparent water-in-air contact angle on scaffolds untreated and treated with NaOH are given in Figure 1. As previously reported [28,29], the contact angle indicates the wettability of the material surface, indicating hydrophilic/hydrophobic characteristics of the material. In general, a contact angle above 90° corresponds to a hydrophobic surface, while a contact angle value below 90° represents a hydrophilic surface. In the untreated case, the values slightly decreased with the addition of pristine graphene, ranging from 96° ± 1.50° (neat PCL) to 84° ± 2.90° (0.50 wt % pristine graphene). Contrary to the common assumption that graphene, as other carbon-based materials, is hydrophobic, these results are in line with recent observations from Munz and co-workers [30]. Those researchers investigated the adhesion and friction properties of single-layer and double-layer graphene using chemical force microscopy with a hydrophobic probe. Results showed a large adhesion force between the probe and double-layer graphene compared to single-layer, showing that double-layer graphene is ideal for hydrophobic applications and single-layer graphene for applications where a hydrophilic surface is required. Figure 1 shows a bar chart with the contact angle values. A statistical difference was observed between pure PCL scaffolds and both 0.50 wt % and 0.78 wt % pristine graphene scaffolds before NaOH treatment, indicating that the hydrophilicity of the surface increased with a small concentration of pristine graphene. After NaOH treatment, neat PCL (61° ± 6.50°), 0.13 wt % (69° ± 6.72°), and 0.50 wt % (67° ± 6.09°) pristine graphene scaffolds had a significant reduction in contact angle, compared to untreated scaffolds, and were statistically different from 0.78 wt % (83° ± 7.06°).

### 2.2. Morphological Evaluation of Scaffolds

**Figure 2:**
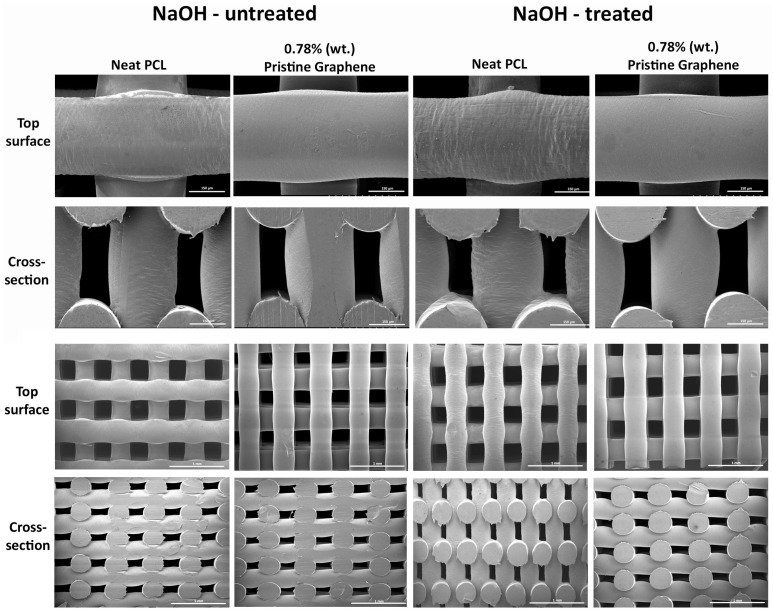
Top surface and cross-section scanning electron microscope images of neat PCL and 0.78 wt % pristine graphene scaffolds treated and untreated with NaOH.

Figure 2 represents the fibre surface of 0.78 wt % pristine graphene and neat PCL scaffolds treated and untreated with 5 M NaOH for 3 h. Results show that, for the NaOH chemical treatment time considered, there is no negative impact on the fibre structure in the produced scaffolds. Similar results were obtained for the other compositions. It is also evident that the produced scaffolds have regular, well-defined, and uniform pore distribution.

### 2.3. Biological Evaluation

**Figure 3:**
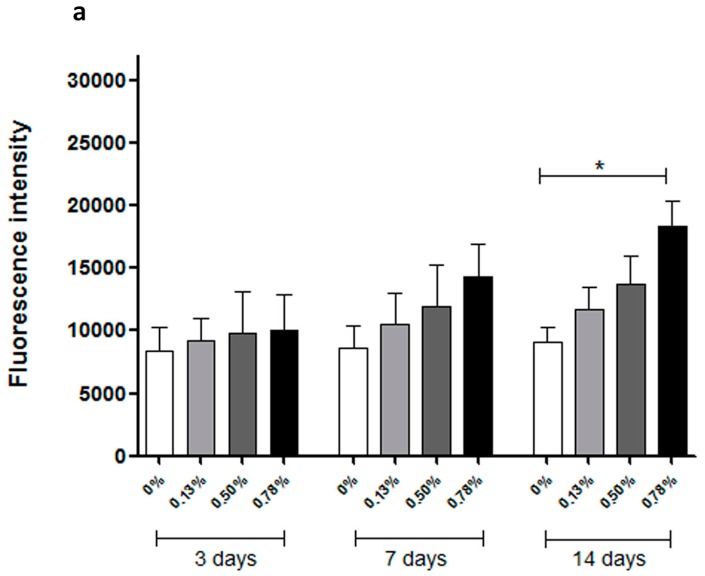

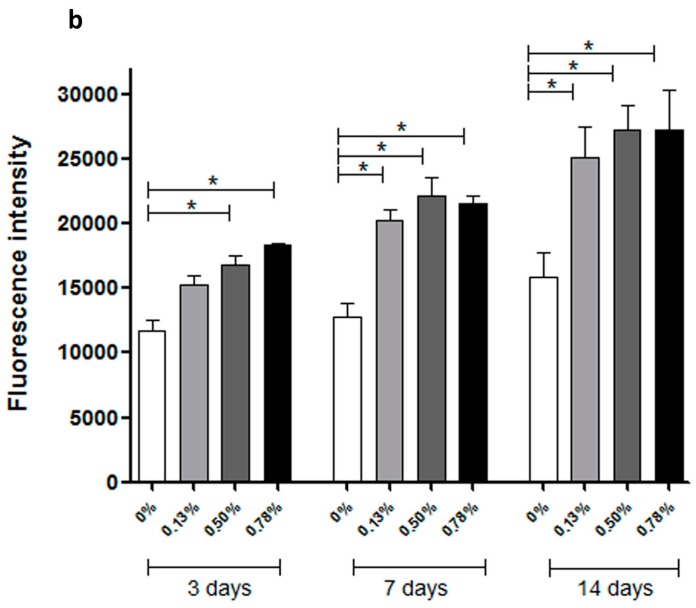
Cell viability/proliferation (Fluorescence intensity) after 3, 7, and 14 days of cell seeding. (**a**) Untreated scaffolds; (**b**) NaOH-treated scaffolds. * Statistical evidence (*p* < 0.05) analysed with a one-way ANOVA and Tukey’s post-hoc test.

Cell viability and proliferation on scaffold samples were assessed using an Alamar Blue assay. Fluorescence intensity is reported in Figure 3. Higher fluorescence intensity corresponds to more metabolically active cells. Comparing the three different time points in Figure 3a or Figure 3b, it can be observed that, for scaffolds both treated and untreated with NaOH, fluorescence intensity increases from one point in time to another, suggesting that scaffolds fabricated with the additive manufacturing system are suitable substrates for cell proliferation. Compared with values at the same point in time in Figure 3a or Figure 3b for untreated scaffolds, the addition of pristine graphene has a positive impact on the biological behaviour of polymer scaffold, but not a significant one. At Day 14, the fluorescence intensity of the 0.78 wt % PCL/pristine graphene scaffold was statistically higher than the neat PCL scaffold, representing a higher cell viability/proliferation rate. For NaOH-treated scaffolds, it can be observed that the addition of pristine graphene had a significant positive impact on cell viability/proliferation. According to statistical analysis, at Day 3, 0.50 wt % and 0.78 wt % scaffolds exhibited greater fluorescence intensity, statistically different from the neat and 0.13 wt % scaffolds, corresponding to higher cell viability/proliferation rates. At Days 7 and 14, all PCL/pristine graphene scaffolds exhibited better biological performance over the neat PCL scaffolds.

Comparing Figure 3a,b, it is evident that, at all three time points, scaffolds treated with NaOH had higher fluorescence intensity than non-treated scaffolds, presenting an improved biological performance. After 14 days, results indicated that, after chemical treatment with NaOH, the improvements on biological behaviour caused by the addition of pristine graphene still exist. For each particular time point in Figure 3b, PCL/pristine graphene scaffolds showed higher fluorescence intensity than neat PCL scaffolds, with cell proliferation rate increasing concomitant to increase graphene addition.

**Figure 4:**
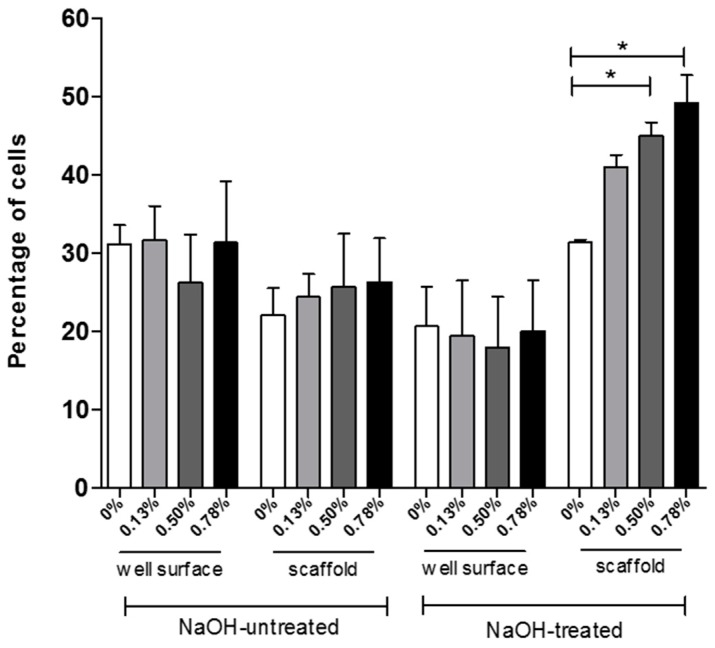
Percentage of cells attached on the well plate surface (cells not attached to the scaffold) and percentage of cells attached on the scaffold after 3 days of cell seeding. * Statistical evidence (*p* < 0.05) analysed with a one-way ANOVA and Tukey’s post-hoc test.

Figure 4 represents the assessment of cells attached on the scaffolds and left on the surface of the well plate. The measurement was performed at the first time point (3 days) after cell seeding, which is a representation of the cell attachment rate. It is evident that scaffold samples treated with NaOH had a higher cell attachment rate than the untreated scaffolds. Scaffolds untreated had around 30% cell attachment, while scaffolds treated with NaOH varied widely among samples. Moreover, 0.50 wt % and 0.78 wt % pristine graphene scaffolds had statistically higher cell attachment rate after 3 days compared to the neat PCL scaffold. Cell attachment is closely related to the surface properties of the scaffolds. The chemical treatment with NaOH leads to enhanced hydrophilicity and more cell attachment when compared to the untreated scaffolds. Furthermore, the addition of pristine graphene (0.50 wt % and 0.78 wt %) improved the cell attachment and proliferation rate even more after three days.

As is evident from Figure 3 and Figure 4, pristine graphene has a significant impact on the biological performance of produced scaffolds, increasing cell attachment and proliferation. This can be attributed to the high surface area, the elastic modulus, and the stiffness of graphene. It is also related to the presence of wrinkles and ripples on graphene, created during the production of graphene [31]. Graphene was also found to be useful as a cellular adhesive, preventing implanted cells from reactive oxygen species (ROS)-mediated cell death, enhancing cell proliferation [32]. 

### 2.4. Cell Attachment and Cell Morphology

**Figure 5:**
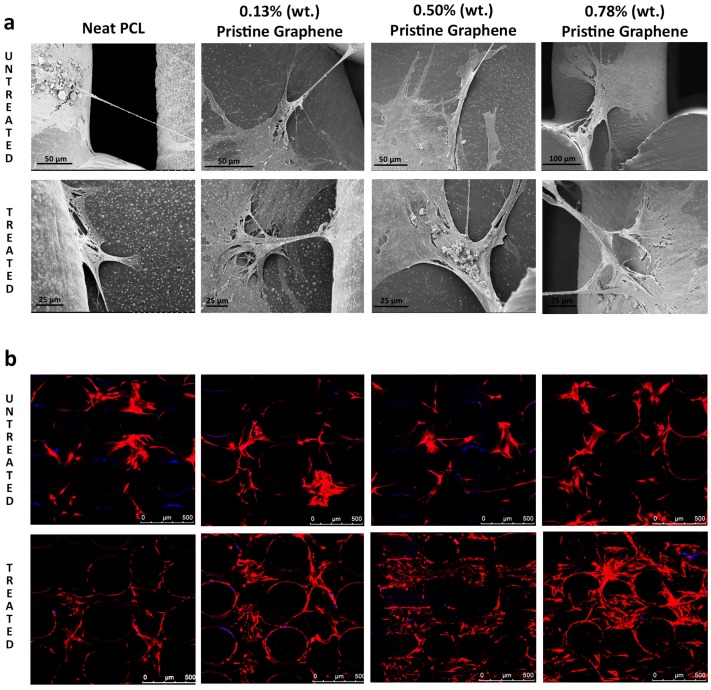
Cells attached on the scaffold. (**a**) SEM images after 21 days culture; (**b**) confocal images after 28 days culture.

Cell attachment and morphology on the scaffolds was assessed via scanning electron microscopy (SEM) and laser confocal microscopy. Extensive cell attachment and cell spreading was observed, as shown in Figure 5. Confluent cell sheets were also observed, with many cells bridging orthogonal scaffold filaments. This indicates that the scaffolds are able to provide a suitable environment and support the growth and proliferation of cells. The confocal images show that cell morphology is maintained (nuclei stained blue, cell membrane red). Qualitatively, for all types of scaffolds, both SEM and confocal images show a greater number of cells present for scaffolds containing pristine graphene. NaOH-treated scaffolds presented higher cell confluency than untreated scaffolds.

## 3. Materials and Methods

### 3.1. Scaffold Fabrication

Poly (*ε*-caprolactone) (*M*_w_ 50,000, Capa 6500, Perstorp, Warrington, UK) and pristine graphene were used to produce scaffolds through a screw-assisted additive biomanufacturing system, the 3D Discovery (RegenHU, Villaz-St-Pierre, Switerzland), as previously reported [28]. PCL/pristine graphene pellets were initially prepared by melt blending in three different pristine graphene concentrations (0.25, 0.50, and 0.75 wt %) [28]. Briefly, pure PCL pellets were heated above 70 °C in a bowl to ensure all material is in a molten state prior to pristine graphene flake addition, at the desired concentrations. The materials were mixed for 15 min to guarantee a homogenously dispersion. After cooling down for 2 h, the blended material was cut into small pellets to be loaded to the screw-assisted additive biomanufacturing system. As previously reported [28], the obtained pristine graphene concentrations are 0.13, 0.50, and 0.78 wt %.

The extrusion-based additive manufacturing technique used in this work allows high reproducibility and good control over scaffold topology (including pore size, pore shape, and pore distribution), which are critical parameters when designing optimised 3D interconnected porous scaffolds [28,33]. The 0°/90° lay-down pattern was adopted to obtain pores with a regular square geometry and a constant filament distance of 680 μm. The optimal combination of processing parameters—melting temperature (90 °C), slice thickness (220 μm), screw rotation velocity (22 rpm), and deposition velocity (20 mm/s)—allows for the filament diameter after extrusion to be close to the desired diameter of 330 μm [28,33]. As previously reported, the presence of pristine graphene at the surface of the scaffold filaments was observed using Raman spectroscopy, and micro Raman mapping showed a uniform distribution of pristine graphene [33].

After fabrication, scaffold samples were cut with fine, double-edged razor blades into small blocks (~11 mm × 11 mm × 6 mm) to fit in a 24-well culture plate.

### 3.2. Surface Modification

Scaffold samples for the NaOH-treated group were processed by soaking in 5 M NaOH for 3 h using 50 mL conical tubes, at room temperature [11]. The scaffolds were washed exhaustively with distilled water and air-dried for 24 h in an incubator at 37 °C.

### 3.3. Apparent Water-in-Air Contact Angle

Static contact angle measurements were performed on produced scaffolds using an OCA 15 (Data Physics) machine using the sessile drop method. Deionised water droplets of ~4 µL were deposited via a motorised syringe at a velocity of 1 µL/s. Five measurements per sample type were performed. The drop shape was recorded with a high speed framing camera. Measurements were performed 20 s after droplet addition.

### 3.4. Morphological Characterisation

Scanning electron microscopy (SEM) was used to investigate the morphology of produced scaffolds treated and untreated with 5 M NaOH. SEM was conducted with a Quanta 200 SEM system, using an accelerating voltage of 3.0 kV.

### 3.5. Cell Culture Studies

In vitro cell culture studies were conducted using Human adipose-derived stem cells (ADSCs) (STEMPRO^®^, Invitrogen, Waltham, MA, USA) ranging from passage 3 to 5. Cells were cultured in T75 tissue culture flasks (Sigma-Aldrich, Dorset, UK) with MesenPRO RS™ Basal Medium (Invitrogen, Waltham, MA, USA) until 80% confluence and harvested by the use of a 0.05% trypsin solution (Invitrogen, Waltham, MA, USA).

#### 3.5.1. Cell Seeding

Scaffolds were sterilised in 70% ethanol for 4 h, rinsed in phosphate buffered saline (PBS) three times, placed in 24-well plates, and air-dried for 24 h in an incubator at 37 °C. The scaffolds were wet with 200 µL of media containing 10% foetal bovine serum (FBS) and kept in standard conditions (37 °C under 5% CO_2_ and 95% humidity) for 2 h prior to cell seeding [11]. Cells were seeded on each scaffold (150 µL of medium containing around 5 × 10^4^ cells). A tissue culture plastic (TCP) control containing the same amount of cells was included for consideration as 100% of cells seeded to evaluate the seeding efficiency after 3 days. The cell-seeded scaffolds and control were incubated at standard conditions (37 °C under 5% CO_2_ and 95% humidity) for 2 h to allow cell attachment, before the addition of 1 mL of fresh basal media [11,34].

#### 3.5.2. Cell Viability/Proliferation

In order to study the cell viability/proliferation and the percentage of cells attached in the scaffolds (cell-seeding efficiency), the Alamar Blue assay (also termed the Resazurin assay) was used (reagents from Sigma-Aldrich, Dorset, UK) [35,36]. Briefly, cell viability/proliferation was measured at 3, 7, and 14 days after cell seeding to PCL and PCL/pristine graphene scaffolds treated and untreated with NaOH. After 3 days of cell seeding, but before the Alamar Blue test, the cell-seeded scaffolds were moved to a new 24-well plate and 1 mL of the Alamar Blue solution was added to each well and the control. Cells attached on the surface of the wells were also quantified on the 3rd time point. The plates were incubated for 4 h under standard conditions. After incubation, 150 µL of each sample was transferred to a 96-well plate, and the fluorescence intensity was measured at 540 nm excitation wavelength and 590 nm emission wavelength with a spectrophotometer (Sunrise, Tecan, Männedorf, Zurich, Switzerland). Experiments were performed three times in duplicate.

#### 3.5.3. Cell Morphology and Attachment

Samples of scaffolds used in the cell viability/proliferation study were kept in culture up to 21 days to assess cell morphology and qualitative attachment to the scaffolds via scanning electron microscopy (SEM). For SEM preparation, scaffolds were fixed with a 3% glutaraldehyde solution (Sigma-Aldrich, UK) for 30 min at room temperature, rinsed twice with PBS, dehydrated with a graded ethanol series (50%, 70%, 80%, 90%, and 100% (twice)), in 50:50 ethanol/hexamethyldisilazane (HMDS, Sigma-Aldrich, Dorset, UK) and then in 100% HMDS (with 10 min exposure at each step), and then air dried for HMDS removal [11]. Thin cross-section layers of each sample (around 1 mm) was cut and platinum-coated for imaging using a Gatan Model 682 Precision Etching Coating System, to an approximate thickness of 7 nm. SEM images were obtained using a Hitachi S300N microscope (Hitachi, Maidenhead, UK).

Cell morphology was further assessed using laser confocal microscopy with scaffolds cultured up to 28 days, with cell membranes and nuclei stained. Samples were removed from the cell culture plate, rinsed twice in PBS, fixed using 4% paraformaldehyde for 40 min, and then washed twice with PBS prior to immersion for 30 min in an immunocytochemistry blocking buffer comprised of 2% goat serum and 1% bovine serum albumin in PBS. Samples were again rinsed twice in PBS. Cell nuclei were stained blue by soaking them in a PBS solution containing Hoescht 33342 (C62249, ThermoFisher, Waltham, MA, USA) at a 2 µM concentration; the cell membranes stained using CellMask™ Orange plasma membrane stain (C10045, ThermoFisher, Waltham, MA, USA) were diluted to the manufacturer recommended level [37,38]. Samples were left in the staining solution for 10 min prior to removal, rinsed twice thoroughly with PBS, and mounted using ProLong^®^ Diamond Antifade (P36962, ThermoFisher, Waltham, MA, USA). Confocal images were obtained on a Leica TCS SP5 (Leica, Milton Keynes, UK) confocal microscope.

### 3.6. Data Analysis

All data were represented as mean ± standard deviation. A one-way analysis of variance (one-way ANOVA) and Tukey’s post-hoc test using GraphPad Prism software was applied. Significance levels were set at *p* < 0.05.

## 4. Conclusions

Scaffolds with filaments containing well dispersed pristine graphene produced by an additive manufacturing system presented good biological behaviour in terms of cell viability and proliferation, making them a good substrate for bone tissue regeneration. The addition of low concentrations of pristine graphene exhibited no cytoxicity, and enhanced cell viability/proliferation.

The addition of pristine graphene, served to moderately reduce the apparent water-in-air contact angle compared to the neat PCL filaments. Chemical treatment with 5 M NaOH further increased the hydrophilicity, leading to better cell attachment and enhanced biological behaviour. Test results also proved that the NaOH treating process did not change the enhancement in biological performance due to the addition of pristine graphene.

It can be concluded that PCL/pristine graphene scaffolds fabricated by an extrusion-based additive manufacturing system could be a promising substrate for bone tissue engineering, and that NaOH chemical treatment could effectively improve the biological behaviour of these composite scaffolds.

## References

[1] Bartolo P.J., Chua C.K., Almeida H.A., Chou S.M., Lim A.S. (2009). Biomanufacturing for tissue engineering: Present and future trends. Virtual Phys. Prototyp..

[2] Bartolo P.J., Kruth J.P., Silva J., Levy G., Malshe A., Rajurkar K., Mitsuishi M., Ciurana J., Leu M. (2012). Biomedical production of implants by additive electro-chemical and physical processes. CIRP Ann. Manuf. Technol..

[3] Vaezi M., Yang S. (2015). Extrusion-based additive manufacturing of PEEK for biomedical applications. Virtual Phys. Prototyp..

[4] Bártolo P.J., Almeida H.A., Rezende R.A., Laoui T., Bidanda B. (2008). Advanced processes to fabricate scaffolds for tissue engineering. Virtual Prototyping & Bio Manufacturing in Medical Applications.

[5] Bártolo P.J., Almeida H., Laoui T. (2009). Rapid prototyping and manufacturing for tissue engineering scaffolds. Int. J. Comput. Appl. Technol..

[6] Dean D., Mott E., Luo X., Busso M., Wang M.O., Vorwald C., Siblani A., Fisher J.P. (2014). Multiple initiators and dyes for continuous Digital Light Processing (cDLP) additive manufacture of resorbable bone tissue engineering scaffolds: A new method and new material to fabricate resorbable scaffold for bone tissue engineering via continuous Digital Light Processing. Virtual Phys. Prototyp..

[7] Wang S., Lee J.M., Yeong W.Y. (2015). Smart hydrogels for 3D bioprinting. Int. J. Bioprint..

[8] Wang M., He J., Liu Y., Li M., Li D., Jin Z. (2015). The trend towards in vivo bioprinting. Int. J. Bioprint..

[9] Lee J.M., Yeong W.Y. (2015). A preliminary model of time-pressure dispensing system for bioprinting based on printing and material parameters: This paper reports a method to predict and control the width of hydrogel filament for bioprinting applications. Virtual Phys. Prototyp..

[10] Khoo Z.X., Teoh J.E., Liu Y., Chua C.K., Yang S., An J., Leong K.F., Yeong W.Y. (2015). 3D printing of smart materials: A review on recent progresses in 4D printing. Virtual Phys. Prototyp..

[11] Caetano G., Violante R., Sant A.B., Murashima A.B., Domingos M., Gibson A., Bártolo P., Frade M.A. (2016). Cellularized versus decellularized scaffolds for bone regeneration. Mater. Lett..

[12] Poh P.S., Hutmacher D.W., Holzapfel B.M., Solanki A.K., Stevens M.M., Woodruff M.A. (2016). In vitro and in vivo bone formation potential of surface calcium phosphate-coated polycaprolactone and polycaprolactone/bioactive glass composite scaffolds. Acta Biomater..

[13] Domingos M., Intranuovo F., Gloria A., Gristina R., Ambrosio L., Bártolo P.J., Favia P. (2013). Improved osteoblast cell affinity on plasma-modified 3-D extruded PCL scaffolds. Acta Biomater..

[14] Sousa I., Mendes A., Pereira R.F., Bártolo P.J. (2014). Collagen surface modified poly (*ε*-caprolactone) scaffolds with improved hydrophilicity and cell adhesion properties. Mater. Lett..

[15] Kuilla T., Bhadra S., Yao D., Kim N.H., Bose S., Lee J.H. (2010). Recent advances in graphene based polymer composites. Prog. Polym. Sci..

[16] Tiaw K.S., Goh S.W., Hong M., Wang Z., Lan B., Teoh S.H. (2005). Laser surface modification of poly (*ε*-caprolactone) (PCL) membrane for tissue engineering applications. Biomaterials.

[17] Yeo A., Wong W.J., Teoh S.H. (2010). Surface modification of PCL-TCP scaffolds in rabbit calvaria defects: Evaluation of scaffold degradation profile, biomechanical properties and bone healing patterns. J. Biomed. Mater. Res. Part A.

[18] Tan P.S., Teoh S.H. (2007). Effect of stiffness of polycaprolactone (PCL) membrane on cell proliferation. Mater. Sci. Eng. C.

[19] Geim A.K., MacDonald A.H. (2007). Graphene: Exploring carbon flatland. Phys. Today.

[20] Si Y., Samulski T. (2008). Synthesis of water soluble graphene. Nano Lett..

[21] Worsley K.A., Ramesh P., Mandal S.K., Niyogi S., Itkis M.E., Haddon R.C. (2007). Soluble graphene derived from graphite fluoride. Chem. Phys. Lett..

[22] Niyogi S., Bekyarova E., Itkis M.E., McWilliams J.L., Hamon M.A., Haddon R.C. (2006). Solution properties of graphite and graphene. J. Am. Chem. Soc..

[23] Liao K.H., Lin Y.S., Macosko C.W., Haynes C.L. (2011). Cytotoxicity of graphene oxide and graphene in human erythrocytes and skin fibroblasts. ACS Appl. Mater. Interfaces.

[24] Wang K., Ruan J., Song H., Zhang J., Wo Y., Guo S., Cui D. (2011). Biocompatibility of graphene oxide. Nanoscale Res. Lett..

[25] Zhang Y., Ali S.F., Dervishi E., Xu Y., Li Z., Casciano D., Biris A.S. (2010). Cytotoxicity effects of graphene and single-wall carbon nanotubes in neural phaeochromocytoma-derived PC12 cells. Acs Nano.

[26] Park S.Y., Park J., Sim S.H., Sung M.G., Kim K.S., Hong B.H., Hong S. (2011). Enhanced differentiation of human neural stem cells into neurons on graphene. Adv. Mater..

[27] Li N., Zhang X., Song Q., Su R., Zhang Q., Kong T., Liu L., Jin G., Tang M., Cheng G. (2011). The promotion of neurite sprouting and outgrowth of mouse hippocampal cells in culture by graphene substrates. Biomaterials.

[28] Wang W., Caetano G.F., Chiang W.H., Braz A.L., Blaker J.J., Frade M.A., Bartolo P.J. (2016). Morphological, mechanical and biological assessment of PCL/pristine graphene scaffolds for bone regeneration. Int. J. Bioprint..

[29] Llorens E., Calderón S., del Valle L.J., Puiggalí J. (2015). Polybiguanide (PHMB) loaded in PLA scaffolds displaying high hydrophobic, biocompatibility and antibacterial properties. Mater. Sci. Eng. C.

[30] Munz M., Glusca C.E., Myers-Ward R.L., Gaskill D.K., Kazakova O. (2015). Thickness-dependent hydrophobicity of epitaxial graphene. ACS Nano.

[31] Lee W.C., Lim C.H.Y.X., Shi H., Tang L.A.L., Wang Y., Lim C.T., Loh K.P. (2011). Origin of enhanced stem cell growth and differentiation on graphene and graphene oxide. ACS Nano.

[32] Kim T.M., Lee T., El-Said W.A., Choi J.W. (2015). Graphene-based materials for stem cell aaplications. Materials.

[33] Wang W., Chiang W.H., Bartolo P.J., Chua C.K., Lau G.K., Moon S.K., Zhang Y.L., Zhou K., Zhou Y.F. (2016). Design, Fabrication and Evaluation of PCL/Graphene Scaffolds for Bone Regeneration, Proceedings of the 2nd International Conference on Progress in Additive Manufacturing, Singapore, 16–19 May 2016.

[34] Caetano G.F., Bártolo P.J., Domingos M., Oliveira C.C., Leite M.N., Frade M.A. (2015). Osteogenic differentiation of adipose-derived mesenchymal stem cells into Polycaprolactone (PCL) scaffold. Procedia Eng..

[35] Zhang H.X., Du G.H., Zhang J.T. (2004). Assay of mitochondrial functions by resazurin in vitro. Acta Pharmacol. Sin..

[36] Vega-Avila E., Pugsley M.K. (2011). An Overview of Colorimetric Assay Methods Used to Assess Survival or Proliferation of Mammalian Cells. Proc. West Pharmacol. Soc..

[37] Parish C.R. (1999). Fluorescent dyes for lymphocyte migration and proliferation studies. Immunol. Cell Boil..

[38] Ortiz de Solorzano C., Malladi R., Lelievre S.A., Lockett S.J. (2001). Segmentation of nuclei and cells using membrane related protein markers. J. Microsc..

